# Electro-clinical features and management of the late stage of Lafora disease

**DOI:** 10.3389/fneur.2022.969297

**Published:** 2022-10-05

**Authors:** Giuseppe d'Orsi, Maria Teresa Di Claudio, Orazio Palumbo, Massimo Carella

**Affiliations:** ^1^Neurology Unit, Fondazione Istituto di Ricovero e Cura a Carattere Scientifico Casa Sollievo della Sofferenza, San Giovanni Rotondo, FG, Italy; ^2^Department of Neurological Sciences, Policlinico Riuniti, University Neurology Unit, Foggia, Italy; ^3^Division of Medical Genetics, Fondazione Istituto di Ricovero e Cura a Carattere Scientifico Casa Sollievo della Sofferenza, San Giovanni Rotondo, FG, Italy

**Keywords:** Lafora disease, late stage, status epilepticus, medical complications, management, electro-clinical features

## Abstract

**Purpose:**

The aim of this study was to elucidate the electro-clinical features and management of the late stage of Lafora disease (LD).

**Methods:**

We investigated the electro-clinical data and medical complications of three LD patients with mutations in *EPM2A* and two in *NHLRC1* genes during the LD late stage.

**Results:**

The late stage emerged after a mean period of 7 ± 1.41 years from the onset of the disease. All patients developed gait ataxia becoming bedbound with severe dementia. Pluri-monthly and drug-resistant myoclonic seizures, and myoclonic absence and tonic–clonic seizures were associated with daily/pluri-daily myoclonus, while the EEG/polygraphic findings showed diffusely slow activity with epileptiform abnormalities, often correlated with myoclonic jerks. Seizure emergencies with motor cluster/status epilepticus and medical complications dominated the clinical picture. In particular, video-EEG/polygraphic recordings disclosed status epilepticus with prominent motor symptoms of different subtypes refractory to IV new anti-seizure medications and responsive in 75% of cases to IV phenytoin. The main complications were dysphagia, aspiration pneumonia, acute respiratory failure, sepsis, immobility, and spasticity with bedsores. A coordinated and multidisciplinary management of the three patients with *EPM2A* mutations has demonstrated a reduction in seizure emergencies, medical complications and days of hospitalization, and a prolongation of the years of disease compared to the two patients with *NHLRC1* mutations.

**Conclusion:**

Status epilepticus with prominent motor symptoms of different subtypes, often responsive to IV phenytoin, and multiple medical complications characterize the LD late stage. An effective management requires a multidisciplinary medical and nursing team, coordinated by an epileptologist with the aim of reducing seizure emergencies and medical complications.

## Introduction

Lafora disease (LD, OMIM# 254780) is an autosomal recessive neurodegenerative disorder caused by loss-of-function mutations in either the laforin glycogen phosphatase gene *(EPM2A)* or the malin ubiquitin E3 ligase *(NHLRC1)* (1, 2). Clinically, LD is an adolescence-onset disease, which results in progressive myoclonic epilepsy (PME) (1, 3). As the disease progresses, LD patients present a rapidly progressive dementia concomitant with refractory or super-refractory status epilepticus (SE), intractable myoclonus, cerebellar ataxia and dysarthria, mutism, and respiratory failure leading to death usually within a several years after the first symptoms (1, 4).

The LD management includes diagnosis and genetic counseling, treatment, and familial support (1). In particular, the recognition of the presenting symptoms (3) allows an early diagnosis with a better genetic counseling and management of affected patients and their families, using emerging personalized treatments that may slow the evolution of the disease (2). In fact, LD is very often misdiagnosed at the onset (1) as juvenile myoclonic epilepsy (JME), a subtype of genetic generalized epilepsy. Tonic–clonic and focal visual seizures followed by myoclonic seizures and action-postural myoclonus together with EEG background slowing with diffuse and occipital epileptiform abnormalities suggest a diagnosis of LD (3). Unverricht–Lundborg disease (ULD) can also be misdiagnosed as JME, particularly at the onset. Like in LD, the symptom that makes the difference in JME is the action myoclonus, which can become clearly evident even many years after the seizure onset (5).

Instead, the management during the evolution of LD especially requires the treatment of non-specific medical complications in bedridden and demented patients, and a social support (1).

In one of the rare LD case series, Tassinari et al. (6) described three evolutive electro-clinical phases: a first phase characterized by tonic–clonic seizures with EEG features similar to those shown for genetic generalized epilepsy; a second phase presenting myoclonus associated with a progressive slowing of the posterior background and diffuse faster and irregular discharges of spike waves; finally, a late phase characterized by dementia and diffusely slow EEG with superimposed fast multiple spikes. We reported the electro-clinical features of the late stage of disease in five LD patients. Since a full and detailed management description lacks, we wish to share our experience with neurologists and other physicians dealing with LD patients in the late phase of the disease.

## Methods

We electro-clinically evaluated five LD patients (three males, two females; mean age: 23.2 ± 2.58 years, median 24, range 20–26) native of the Apulia Region in Southern Italy. The diagnosis was made by genetic analysis. In particular, the next-generation sequencing analysis disclosed the presence of mutations in the *EPM2A* gene in three patients and in the *NHLRC1* gene in the remaining two patients (3). All LD patients underwent brain magnetic resonance imaging (MRI; 1.5-Tesla System), which revealed moderate and diffuse cerebral and cerebellar atrophy after 3.3 years (range 2–6) from the onset of the disease.

The parameters of the video-EEG/polygraphic recordings included video-EEG (electrodes were placed based on the 10–20 International System with bipolar montage); an electromyogram (EMG) of both deltoid muscles, the right and left flexor and extensor muscles of the hand, and both tibialis anterior muscles; an EKG; and thoracic respiration (monitored using a strain gauge). Polygraphic EMG signals were recorded using pairs of surface electrodes with standard belly-tendon placement. Signals were acquired digitally (sampling frequency: 512 Hz; band-pass filters: 1.6–210 Hz; Nihon Kohden, Tokyo, Japan). The relationship between EEG and EMG bursts (myoclonus) was analyzed by applying jerk-locked back-averaging. Myoclonus severity was scored using a simplified myoclonus rating scale (7): 0 no myoclonus; 1 minor myoclonus, no interference with daily living; 2 mild myoclonus, interference with fine movements and/or speech, no interference with walking; 3 moderate myoclonus, patient still able to walk without support; 4 moderate-to-severe myoclonus, patient able to stand, unable to walk without support; and 5 severe myoclonus, patient wheelchair-bound or bedridden.

Status epilepticus was diagnosed according to the definition and classification proposed by the ILAE Task Force (8). We considered a response to an anti-seizure medication (ASM) when it was the last drug administered prior to the clinical and/or EEG resolution of seizures, and SE did not recur during hospital observation.

The local Ethics Committee on human experimentation approved the study, and a written informed consent was obtained from the patients' relatives.

## Results

The mean age at LD onset was 12 ± 1.3 years (median 13, range 10–14). Generalized tonic–clonic seizures and focal visual seizures to bilateral tonic–clonic seizures at the onset were sporadic and responsive to ASM monotherapy. Subsequently, after a period ranging from a few to 12 months, patients presented monthly with myoclonic jerks often occurring upon awakening. At the time of the first EEG, background activity was mild and diffusely slow and associated with sporadic diffuse spike-and-wave (SW) or polyspike-and-wave (PSW) abnormalities. After 2 years from the onset of LD, a worsening of the epilepsy and myoclonus associated with a gradual onset of dementia and cerebellar signs (severe gait ataxia with incoordination) emerged. All patients presented daily with drug-resistant myoclonic multifocal jerks precipitated by movements (mean myoclonus severity score was 3.4), while tonic–clonic seizures occurred weekly/pluri-monthly. The EEG background activity slowed further, with diffuse and faster discharges of SW/PSW during awakening and disorganized sleep.

### The late stage

#### Electro-clinical findings

The patients progressively reached this late stage at a mean age of 19.6 ± 1.5 years (median 19, range 18–22) and with a mean period of 7 ± 1.41 years (median 6, range 6–9) from the onset of epilepsy.

Table 1 summarizes the patients' clinical features.

**Table 1:** Clinical features of the late stage of Lafora disease.

**Pt**	**Genetic features**	**Age LD Onset (years), Sex**	**Age LD late stage onset (years)**	**Seizure type**	**Status epilepticus type**	**Myoclonus score**	**Dementia**	**Medical complic ations**	**Disease duration (years)**	**Conditions at last follow-up**
1	EPM2A: c.721C>T p.(Arg241*)	13/F	19	Myoclonic, tonic-clonic	Myoclonic, myoclonic-tonic; NCSE	5	Severe	Dysphagia, aspiration pneumonia, bedsores.	8	Mute and bedridden, with PEG and tracheostomy
2	EPM2A: c.721C>T p.(Arg241*)	13/M	22	Myoclonic, tonic-clonic	Myoclonic, myoclonic-tonic; focal motor; tonic; NCSE	5	Severe	Dysphagia, aspiration pneumonia.	12	Mute and bedridden, with PEG. Death at age 25 from pneumonia
3	EPM2A: c.721C>T p.(Arg241*)	10/M	18	Myoclonic, tonic-clonic	Myoclonic; myoclonic-tonic	5	Severe	Dysphagia, aspiration pneumonia, bedsores	16	Mute and bedridden, with PEG. Death at age 26 from pneumonia
4	NHLRC1: c.992del p.(Gly331Glufs*3)	13/F	19	Tonic-clonic	–	5	Severe	Dysphagia, aspiration pneumonia	11	Mute and bedridden, with PEG. Death at age 24 from SUDEP
5	NHLRC1: c.992del p.(Gly331Glufs*3)	14/M	20	Myoclonic tonic-clonic	Tonic-clonic	4	Moderate	Dysphagia, aspiration pneumonia	6	Moderate ataxia, limited interaction. Death at age 20 from sepsis

LD, Lafora disease; F, female; M, male; PEG, percutaneous endoscopic gastrostomy; NCSE, Non-convulsive SE with coma.

Refractory/super-refractory SE and/or pneumonia *ab ingestis* with percutaneous endoscopic gastrostomy (PEG)/tracheostomy placement represented in all patients the transition to the late stage of disease.

**Figure 1:**
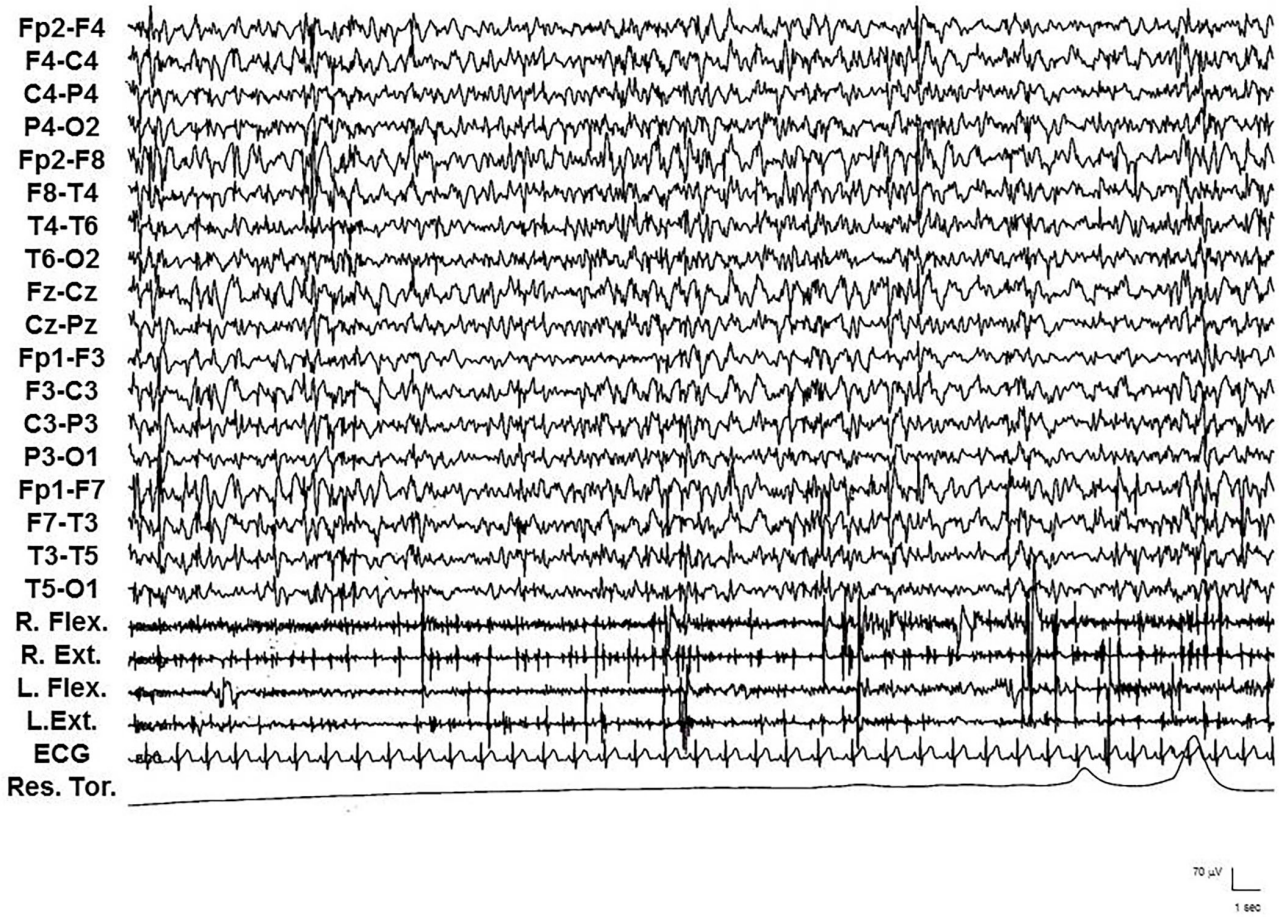
Video-EEG/polygraphic features of interictal pattern in patient 2 during the late stage. Repetitive, multifocal, asymmetrical and asynchronous, rhythmic and arrhythmic, myoclonic jerks associated with diffuse, multifocal, and faster discharges of SW/PSW.

This phase was characterized by a further worsening of the neurological picture, and all five patients were bedbound and had severe mental impairment (MOCA< 10). After 6–10 years from the epilepsy onset, pluri-monthly and drug-resistant myoclonic seizures, and myoclonic absence and tonic–clonic seizures were associated with daily/pluri-daily myoclonic jerks. The mean myoclonus severity score was 4.8. Diffusely slow EEG with diffuse and multifocal SW/PSW discharges, photosensitivity, and sequences of myoclonic jerks often, but not always, associated with epileptiform abnormalities, characterized the EEG/polygraphic findings (see Figure 1).

The mean follow-up duration after epilepsy onset was 10.6 ± 3.84 years (median 11, range 6–16). Patient 1 was bedbound with gastrostomy and tracheostomy, severe mental impairment (MOCA <10), and complete dependence on others for activities of daily living; the disease duration was 8 years at last follow-up. Patient 2 died of pneumonia, and the disease duration was 12 years; he was bedbound with gastrostomy. Patient 3 died of pneumonia, the duration of symptoms was 16 years; he was bedbound with gastrostomy. Patient 4 died of probable sudden unexpected death in epilepsy (SUDEP); she was bedbound with gastrostomy; the duration of symptoms was 11 years. Patient 5 died of sepsis associated with super-refractory SE; he showed moderate mental impairment and moderate gait ataxia, complete dependence on other people for activities of daily living; the duration of symptoms was 6 years.

This final phase was associated with multiple medical complications. All patients experienced poor nutritional intake and dysphagia with the administration of food and fluids *via* a nasogastric tube and, subsequently, *via* a PEG. Other common complications included aspiration pneumonia, acute respiratory failure with tracheostomy placement, sepsis, immobility, and spasticity, which resulted in a higher rate of pressure ulcers. Based on the local availability of devices and the parents' and caregivers' abilities, LD patients were kept at home and followed, by means of an Internet connection, by our Epilepsy team, the medical and the nursing team working in the hospital and in the home setting, and the local caregiving structure. Hospitalization was limited to seizure and medical emergencies not treatable in the home setting.

In particular, this coordinated and multidisciplinary management was possible exclusively in patients 1, 2, and 3 with *EPM2A* mutations, with a consequent reduction not only in seizure emergencies but also in medical complications and days of hospitalization, and a prolongation of the years of disease compared to patients 4–5 with *NHLRC1* mutations.

#### Status epilepticus

Status epilepticus was documented in patients 1, 2, 3, and 5 and emerged at a mean age of 21 ± 1.82 years (median 21, range 19–23), at a mean period of 8.5 ± 3.31 years (median 7.5, range 6–13) after the onset of epilepsy (see Tables 1 and 2).

**Table 2:** Phenitoin in status epilepticus during the late stage of Lafora disease.

**Pt**	**Therapy**	**Status epilepticus type**	**SE type/number of SE treated with IV PHT**	**Treatment before IV PHT**	**Latency (h) onset SE—PHT (median)**	**Success rate**	**Time (h) to success after IV PHT (median)**	**Dosage**	**Adverse events**
1	LEV + VPA + PER + CLZ	Myoclonic, myoclonic-tonic; NCSE	Myoclonic-Tonic/2 NCSE/1	DZP, LCM	24	3/3	3	15–20 mg/kg	Hypotension
2	LEV + VPA + PB	Myoclonic, myoclonic-tonic; focal motor; tonic; NCSE	Myoclonic-tonic/1 focal motor/1 tonic/1 NCSE/1	DZP, LCM, BRV	27	3/4	2	15 mg/kg	None reported
3	VPA + PB + CLZ + PER	Myoclonic; myoclonic-tonic	Myoclonic-tonic/1	DZP, LCM, LEV	30	1/1	2	15 mg/Kg	None reported
5	PER + ZNS + LEV + CLZ	Tonic-clonic	Tonic-clonic/1	DZP, LCM, VPA, TPM	72	0/1	–	15–20 mg/Kg	None reported

PHT, phenytoin; LD, Lafora disease; NCSE, non-convulsive SE with coma; DZP, diazepam; LEV, levetiracetam; VPA, valproate; LCM, lacosamide; PER, perampanel; CLZ, clonazepam; PB, phenobarbital; ZNS, zonisamide; TPM, topiramate; BRV, brivaracetam; SE, status epilepticus.

**Figure 2:**
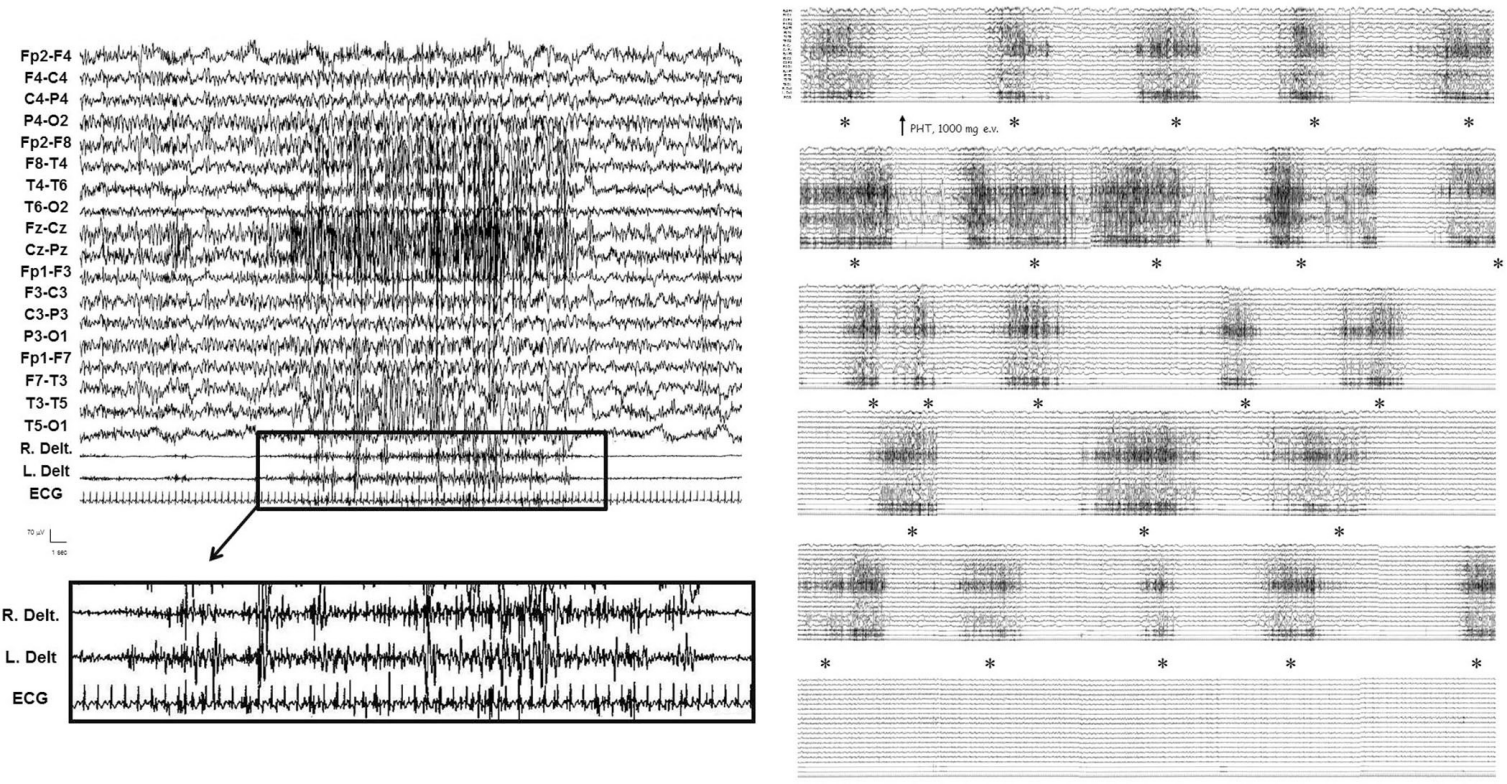
Video-EEG/polygraphic features of myoclonic–tonic SE in patient 1 during the late stage. From the EEG/EMG point of view (see on the left), corresponding to the diffuse and fast paroxysms, myoclonic jerks mainly involving arms, superimposed on a tonic muscular enhancement, appeared; loss of awareness persisted between myoclonic–tonic events. IV diazepam 10 mg, IV lacosamide 400 mg, and IV levetiracetam 3,000 mg were ineffective. Myoclonic–tonic SE resolved after IV phenytoin 1,000 mg in 30 min (see on the right). The asterisks indicate the gradual control of myoclonic–tonic episodes after IV phenytoin.

**Figure 3:**
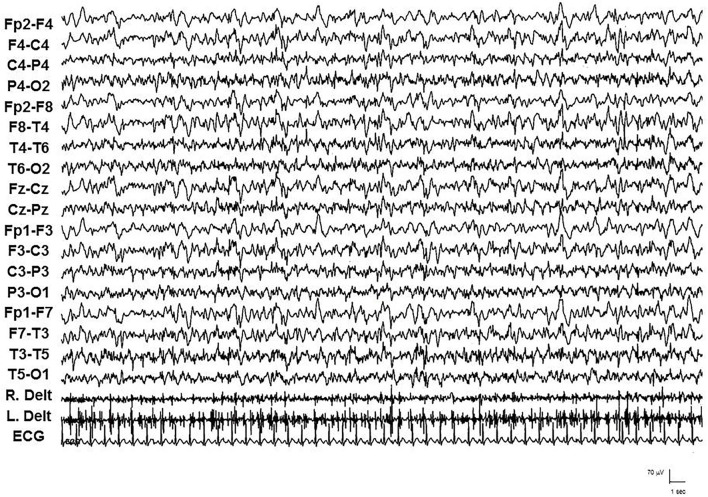
Video-EEG/polygraphic features of focal motor SE in patient 2 during the late stage, with continuous myoclonic jerks in the left arm.

Status epilepticus with prominent motor symptoms is the main form. Different subtypes were documented and often in the same patient: myoclonic, myoclonic–tonic, tonic–clonic, focal motor, and tonic. Myoclonic SE was characterized by repetitive, multifocal, usually asymmetrical and asynchronous, rhythmic and especially arrhythmic myoclonic jerks, associated with diffuse, multifocal, and faster discharges of SW/PSW. IV diazepam (DZP) (10 mg in bolus) or IV delorazepam (2 mg diluted in 100 ml of normal saline), often followed by IV levetiracetam (LEV) (1,500 mg diluted in 100 ml of normal saline), usually resolved SE, particularly when administered at the onset of SE. When particularly massive, myoclonic jerks were intermixed with the increase of muscle tonus and breathing difficulties, establishing a myoclonic–tonic SE. The EEG showed a closely recurring activity of diffuse SW/PSW paroxysms, mainly involving the anterior regions, intermingled with diffuse and brief trains of polyspikes. From the electromyographic point of view, corresponding to the diffuse paroxysms, myoclonic jerks rhythmic and arrhythmic, mainly involving arms, superimposed on a tonic muscular enhancement, appeared (see Figure 2). Because ictal and clinic EEGs were in part similar to those of the myoclonic SE, myoclonic–tonic SE was often confused with myoclonic or tonic–clonic SE by physicians who recorded the medical charts of the patients, and only polygraphic studies disclosed the appropriate diagnosis of the SE type. Moreover, associated with a myoclonic–tonic SE, in one patient a focal motor SE (see Figure 3) and a tonic SE were also observed, sometimes misdiagnosed with tonic–clonic SE.

We documented, under video-EEG/polygraphy and continuous cardiorespiratory monitoring, nine episodes of myoclonic–tonic SE in three patients, one episode of focal motor SE, and one episode of tonic SE in another patient. IV lacosamide (LCM) (200–400 mg) was usually used after IV DZP (10–20 mg), and a transitory, but incomplete, response emerged. Subsequently, IV LEV (2,000–3,000 mg), IV valproate (VPA) (2,000–3,000 mg), and IV brivaracetam (BRV) (200 mg) were used and failed to terminate SE. Finally, 15 mg/kg phenytoin (PHT) was administered after a median time of 1.2 days (range: 1–3) from SE onset; PHT terminated SE in 75% of cases within 2 h after administration. In patients 1, 2, and 3, oral PHT (10 mg/kg) at home prevented clustering of seizures; moreover, IV PHT (15–20 mg/Kg) was utilized as a first-line drug for treatment during other episodes of myoclonic–tonic cluster or SE in the home setting with complete resolution.

Finally, a super-refractory (continuous infusion of midazolam, thiopental, and propofol) tonic–clonic SE associated with sepsis appeared in patient 5.

Non-convulsive SE with coma (“subtle” SE) was exclusively observed in two patients after SE with prominent motor symptoms, with resolution after IV PHT bolus.

## Discussion

The last stage of LD is characterized by rapidly progressive dementia concomitant with drug-resistant epilepsy and refractory or super-refractory SE, intractable action-sensitive and stimulus-sensitive myoclonus, cerebellar ataxia and dysarthria, mutism, and respiratory failure leading to death usually within a several years after the first symptoms (1, 4). In our LD patients, the progressive and fatal evolution was confirmed. At a mean period of 7 ± 1.41 years (median 6, range 6–9) from the onset of epilepsy, a further worsening of the neurological picture appeared, with pluri-monthly and drug-resistant myoclonic seizures, myoclonic absence and tonic–clonic seizures, and refractory/super-refractory SE. Diffusely slow EEG with superimposed diffuse and multifocal SW/PSW discharges, photosensitivity, and sequences of myoclonic jerks often, but not always, associated with epileptiform abnormalities, characterized the EEG/polygraphic findings. All patients developed gait ataxia becoming bedbound, severe dementia, and multiple medical complications. By the time of the last follow-up, three patients had died of multiple medical complications associated with refractory/super-refractory SE, one of SUDEP, while one is bedbound with multiple types of drug-resistant seizures. Although progressive and fatal evolution of LD has been reported and confirmed by our study, full data on management and emergency treatment lack. Turnbull et al. (1) suggest that the final progression of the disease more commonly involves non-specific complications, infectious, or otherwise, in bedridden and demented patients, no longer accompanied by episodes of refractory convulsive SE.

In our experience, a combined management either of seizure emergencies and multiple medical complications in bedridden patients is mandatory during the last phase of the disease, and it affects the final outcome.

### Seizure emergencies

With the progression of LD, seizure chronic treatment progresses to polytherapy with a three to five drug combination, and new ASMs have especially changed the tolerability, but not the overall LD evolution (1, 9). Instead, during seizure emergencies, that is, myoclonic clusters or myoclonic SE, chronic treatment should not be abruptly changed, and emergency therapy includes IV benzodiazepines, IV VPA, and IV LEV (1, 9).

After a full video-EEG/polygraphic study, we observed that SE with prominent motor symptoms is the main form in the late stage of LD and, especially, that myoclonic SE is only one of the different motor SE subtypes.

The documentation of multiple types of SE with prominent motor symptoms, often confused or diagnosed as exclusively myoclonic SE, has had important therapeutic repercussions. In fact, only myoclonic cluster and myoclonic SE have responded to isolated IV BZD or IV BZD followed by IV LEV, especially when administered in the early phases of the emergencies. The other non-exclusively myoclonic subtypes, even if quickly identified and treated, did not have or had a limited and transient response to BZD and new ASMs, whereas IV PHT made it possible to control the motor non-exclusively myoclonic SE in 75% of cases, above all avoiding access to the ICU. Moreover, after the acute phase in the hospital setting, clusters or motor SE which reoccurred in the home setting were treated with IV BZD, for a limited period, and especially with IV PHT with a rapid and complete control, without further hospitalizations.

Moreover, in three patients, we observed the efficacy of oral PHT (10 mg/kg) at home in preventing clustering of seizures, thus SE and hospital stays, while IV PHT (15–20 mg/Kg) was utilized as a first-line drug for treatment during other episodes of myoclonic–tonic cluster or SE in the home setting with complete resolution.

Therefore, we propose the use of IV BZD or IV BZD followed by IV LEV for myoclonic SE, while IV BZD followed by IV PHT or IV PHT for the motor non-exclusively myoclonic SE.

The efficacy of PHT was previously reported in late-stage PMEs (5, 10, 11). In particular, Miyara et al. (10) suggested the efficacy of IV PHT as the treatment of choice for patients with different PMEs (late infantile type neuronal ceroid lipofuscinosis, MELAS/MERRF overlap, Gaucher disease type 3, DRPLA, and degenerative PME) at late stages. Therefore, we suggest that IV PHT, usually contraindicated for PMEs which may increase myoclonic jerks and myoclonic SE, should be used to treat motor non-exclusively myoclonic SE in late-stage LD and PMEs.

Finally, given the fact that PHT is one of the few sodium channel blockers targeting only the sodium channel and no other molecular targets in the brain (12), the favorable response to PHT in SE as well as in the prevention thereof might suggest a possible role of the sodium channel in action potential propagation and spread in LD patients with seizure emergencies.

### Medical complications

Management of medical complications is at least as important as seizure emergency treatment. In fact, our patients experienced poor nutritional intake and dysphagia with PEG placement, aspiration pneumonia, acute respiratory failure with tracheostomy placement, sepsis, immobility, and spasticity with bedsores. Therefore, we coordinated a multidisciplinary hospital medical and nursing team consisting of an epileptologist/neurologist, a gastroenterologist, an infectiologist, a surgeon, an otolaryngologist, and an anesthesiologist. The aim of this team was to prevent and treat multiple complications, such as aspiration pneumonia and the consequences of malnutrition, providing remote assistance to primary care physicians and local care facilities in the home setting and avoiding repetitive hospitalizations. Physical therapy was also continued in this late stage to maintain a good overall muscular condition, treat spasticity, and prevent medical complications. Finally, psychological and social support was very important, especially for LD parents and caregivers, which should receive professional and constant support. According to our experience, during the LD late stage, patients should be maintained at home and followed by means of a continuous connection between the reference specialized epilepsy team and the local caregiving facilities. Hospitalization should be limited to seizure and medical emergencies not treatable in the home setting.

A few studies (13, 14) demonstrated that patients with NHLRC1 mutations, compared to patients with EPM2A mutations, tended to have a slightly milder clinical course, a later onset, and a slower progression.

However, the coordinated and multidisciplinary management exclusively of three patients with EPM2A mutations has demonstrated a reduction not only in seizures emergencies, but also in medical complications and days of hospitalization, and a prolongation of the years of disease compared to the two patients with NHLRC1 mutations.

In conclusion, an effective management of the late stage of LD requires a multidisciplinary network of professionals working both in the home setting and in specialized hospitals coordinated by specialized epilepsy teams, with active involvement of parents and caregivers.

The role of the epileptologists is central and delicate. They are also emotionally and psychologically involved in the management of such a devastating and terminal pathology and will have to guarantee the complicated management of seizure emergencies and supervise the multiple medical complications with the aim of reaching a better management in the final phase of the disease.

## Data availability statement

The datasets presented in this study can be found in online repositories. The name of the repository and accession numbers can be found below: Leiden Open Variation Database (LOVD), https://www.lovd.nl/, Individuals #00301461, #00303263, #00413859, #00413865 and #00413871.

## Ethics statement

Ethical review and approval was not required for the study on human participants in accordance with the local legislation and institutional requirements. Written informed consent to participate in this study was provided by the participants' legal guardian/next of kin.

## Lafora multidisciplinary team

Leonardo De Gennaro, Luigi Auciello, Antonio Ianzano, Melina Bisceglia, Leonardo Piemontese, Rachele Tenace, Pio Guerra, Alessandra Carbone, Giovanni Grumo, Ciro Gisoldo, Michela Colletta, Veronica Marcolongo, Mattea Pastino, Marianna Savella, Antonia Giordano, Alessandra Lalla, Annarita Sabetta, Carlo Avolio, Marina Cela, Anna Carretta, Gianluigi Grilli, Pietro Palumbo, Ester Di Muro, Mario Benvenuto, and Graziana Nesta.

## Author contributions

Gd'O: study concept and design, supervision, writing of the draft, and final revision. MD, OP, MC, and Lafora Multidisciplinary Team: full text review, analysis and interpretation. All authors contributed to the article and approved the submitted version.

## Funding

This work has been funded by Italian Minister of Health, Ricerca Corrente program 2022-2024 and Ricerca Corrente Reti.

## Conflict of interest

The authors declare that the research was conducted in the absence of any commercial or financial relationships that could be construed as a potential conflict of interest.

## Publisher's note

All claims expressed in this article are solely those of the authors and do not necessarily represent those of their affiliated organizations, or those of the publisher, the editors and the reviewers. Any product that may be evaluated in this article, or claim that may be made by its manufacturer, is not guaranteed or endorsed by the publisher.
